# Radiomics analysis of contrast-enhanced computerized tomography for differentiation of gastric schwannomas from gastric gastrointestinal stromal tumors

**DOI:** 10.1007/s00432-023-05545-w

**Published:** 2024-02-09

**Authors:** Cui Zhang, Chongwei Wang, Guoqun Mao, Guohua Cheng, Hongli Ji, Linyang He, Yang Yang, Hongjie Hu, Jian Wang

**Affiliations:** 1 Department of Radiology, TongDe Hospital of ZheJiang Province, No. 234, Gucui Road, Hangzhou, 310013 Zhejiang China; 2 Department of Pathology, Sir Run Run Shaw Hospital, Zhejiang University School of Medicine, Hangzhou, Zhejiang China; 3 Jianpei Technology, Hangzhou, Zhejiang China; 4 Department of Radiology, The First Affiliated Hospital of Bengbu Medical College, Bengbu, Anhui China; 5 Department of Radiology, Sir Run Run Shaw Hospital, Zhejiang University School of Medicine, Hangzhou, Zhejiang China

**Keywords:** Radiomics, Schwannoma, Gastrointestinal stromal tumor, Computed tomography

## Abstract

**Purpose:**

To assess the performance of radiomics-based analysis of contrast-enhanced computerized tomography (CE-CT) images for distinguishing GS from gastric GIST.

**Methods:**

Forty-nine patients with GS and two hundred fifty-three with GIST were enrolled in this retrospective study. CT features were evaluated by two associate chief radiologists. Radiomics features were extracted from portal venous phase images using Pyradiomics software. A non-radiomics dataset (combination of clinical characteristics and radiologist-determined CT features) and a radiomics dataset were used to build stepwise logistic regression and least absolute shrinkage and selection operator (LASSO) logistic regression models, respectively. Model performance was evaluated according to sensitivity, specificity, accuracy, and receiver operating characteristic (ROC) curve, and Delong’s test was applied to compare the area under the curve (AUC) between different models.

**Results:**

A total of 1223 radiomics features were extracted from portal venous phase images. After reducing dimensions by calculating Pearson correlation coefficients (PCCs), 20 radiomics features, 20 clinical characteristics + CT features were used to build the models, respectively. The AUC values for the models using radiomics features and those using clinical features were more than 0.900 for both the training and validation groups. There were no significant differences in predictive performance between the radiomic and clinical data models according to Delong’s test.

**Conclusion:**

A radiomics-based model applied to CE-CT images showed comparable predictive performance to senior physicians in the differentiation of GS from GIST.

**Electronic Supplementary Material:**

The online version of this article (10.1007/s00432-023-05545-w) contains supplementary material, which is available to authorized users.

## Introduction

Gastric schwannoma (GS) is usually a benign, neurogenic, and mesenchymal neoplasm derived from the Schwann cells of the Auerbachs nerve plexus, with an incidence rate of 2–17% (Choi et al. 2012). Gastric gastrointestinal tumors (GISTs) originating from the interstitial cells of Cajal have the highest incidence rate among mesenchymal tumors, accounting for 60–70% of occurrences (Gao et al. 2017; Tsuboi et al. 2021). GSs and GISTs share a similar affected population, clinical symptoms, and even computed tomography (CT) imaging characteristics, particularly in large (> 5 cm) tumors (Hong et al. 2008; Ji et al. 2015; Levy et al. 2003; Wang et al. 2019a, b). However, the treatment strategies and prognoses for these two tumors differ substantially. GS, which is almost always a benign tumor, has an excellent prognosis after surgery (Cai et al. 2016). By contrast, GISTs appear potentially malignant and require complete excision. Furthermore, high-risk GISTs should receive imatinib treatment as adjuvant or neoadjuvant therapy (Casali et al. 2022; Li et al. 2017). It is of great clinical significance to accurately distinguish these two tumors preoperatively.

Numerous studies on the identification of GISTs and GSs on contrast-enhanced (CE)-CT images have been reported (Choi et al. 2012, 2014; He et al. 2017; Liu et al. 2017; Wang et al. 2021; Xu et al. 2022). We previously evaluated five machine learning models to identify the two tumor types on CT image analysis and found that logistic regression classification with six CT features provided the best differentiation of GSs from GISTs, with a diagnostic efficiency greater than traditional multivariate analysis (Wang et al. 2021). Nevertheless, although the abovementioned studies were of value in distinguishing the two kinds of tumors, they all focused on conventional image features recognized by the naked eye and lacked radiomics analysis, thereby relying heavily on the observer’s professional level.

Radiomics features of tumors, which are related to the histopathology and physiology of tumors, can quantitatively and objectively evaluate tumor heterogeneity (Liu et al. 2019). Compared with traditional image evaluation, radiomics features can more comprehensively, objectively, and accurately reflect the nature of a lesion, and permit the establishment of a radiomics database for in-depth quantitative research (Gillies et al. 2016). Radiomics-based analysis has been widely applied to various diseases including the differentiation of benign and malignant lung nodules (Zhuo et al. 2021), classification of liver lesions (Wei et al. 2020), gastric cancer staging (Wang et al. 2020; Liu et al. 2020), determination of malignancy (Zhang et al. 2020; Chen et al. 2019, 2021; Wang et al. 2019a), recurrence (Ao et al. 2021), and Ki-67 expression prediction (Feng et al. 2022) of GISTs.

In this study, CT radiomics features together with clinical data and conventional image features were used to build two kinds of models to distinguish GSs from GISTs: a stepwise logistic regression model and a least absolute shrinkage and selection operator (LASSO) logistic regression model.

## Methods

### Patients

**Fig. 1:**
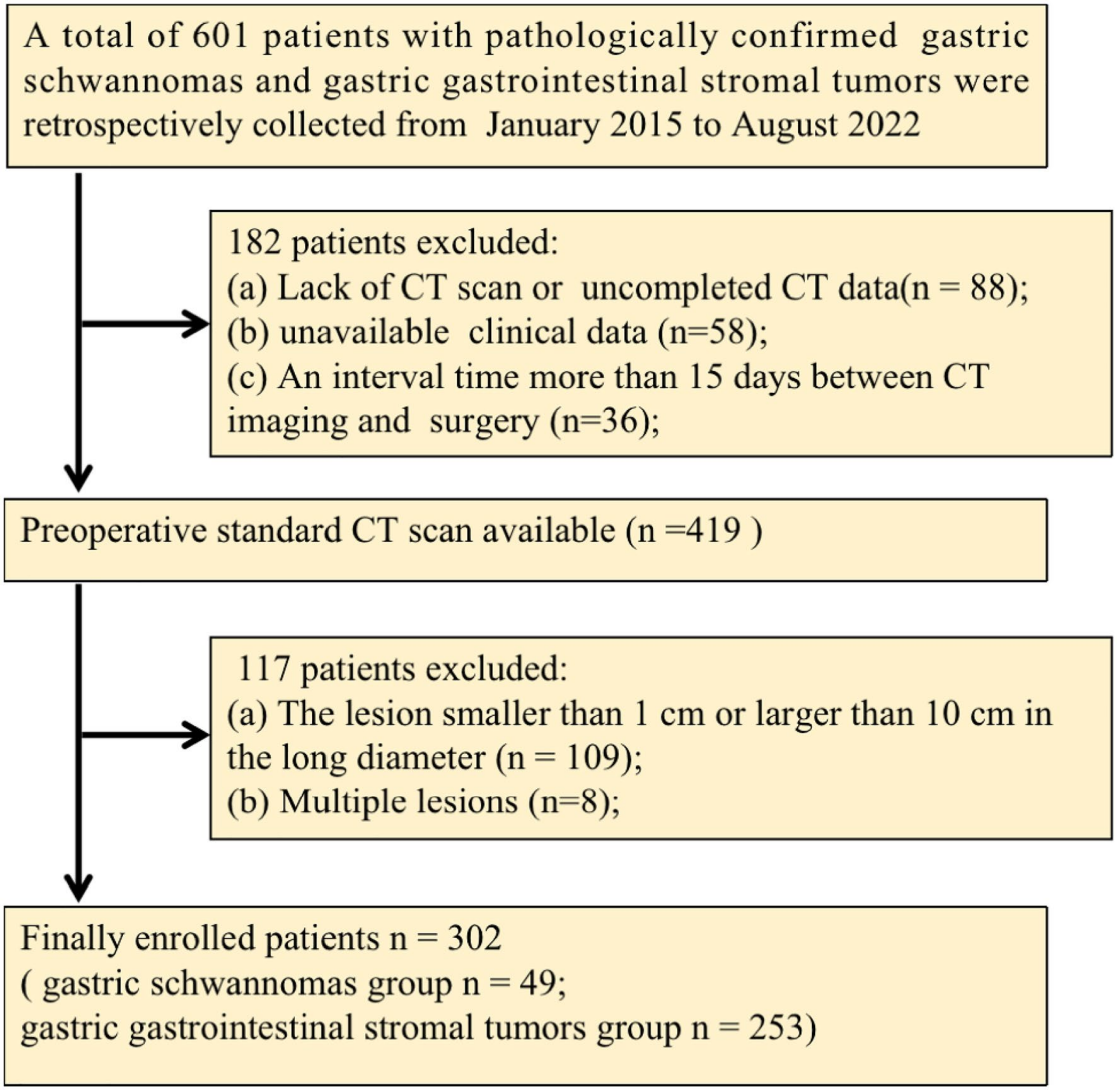
Process of the patients enrolling

This retrospective study received institutional review board and ethics committee approval from Tongde Hospital of Zhejiang Province (Approval No. 2021-040) and informed consent was waived by the ethics review boards. Patients from two centers (Center 1: Tongde Hospital of Zhejiang Province, Center 2: Sir Run Run Shaw Hospital) with either of the two tumor types between January 2015 and August 2022 were identified. The inclusion criteria were: (1) operation-confirmed diagnosis of solitary GIST or schwannoma of the stomach; (2) available detailed clinical data and CE-CT images (with unenhanced, arterial, and portal phases); (3) an interval time within 15 days between CT imaging and surgery; and (4) a lesion larger than 1 cm and smaller than 10 cm in the long diameter. The exclusion criteria were as follows: (1) lack of CT scan or uncompleted CT data; (2) unavailable clinical data; (3) an interval time more than 15 days between CT scan and surgery; (4) the lesion smaller than 1 cm or larger than 10 cm in the long diameter; and (5) multiple lesions. Process of the patients enrolling is shown in Fig. 1. The final study series consisted of 49 patients with GS (16 men and 33 women; mean age, 56.51 ± 10.34 years) and 253 patients with GIST (131 men and 122 women; mean age, 59.56 ± 11.64 years). From these, 211 patients from Center 1 were assigned to a training cohort and 91 patients from Center 2 to a validation cohort. The data for each patient included non-radiomics dataset (consisting of clinical baseline characteristics and CT features) and radiomics dataset.

### CT scan protocols

All patients were requested to drink 800–1000 mL of water on an empty stomach to attain sufficient gastric distension before CT examination. Two 64-slice spiral CT scanners (Siemens Healthineers, Forchheim, Germany; or Philips Medical Systems, Cleveland, OH, USA) were used for the abdominal CE-CT examinations. The CT parameters were: tube voltage, 120 kV; tube current, 150–250 mA; tube rotation time, 0.5 s; detector collimation, 64 × 0.625 mm; field of view, 350 × 350 mm; section thickness, 5 mm; and reconstruction interval, 1–1.5 mm. After a routine unenhanced scan, contrast material was injected at a dose of 1.0 mL/kg body weight at a rate of 3–4 mL/s, and arterial and portal venous phase imaging were acquired at 30–40 s and 60–70 s after injection.

### Image analysis

All CT images were independently retrospectively reviewed and evaluated by two associate chief radiologists (Radiologist 1 with 13 and Radiologist 2 with 15 years of experience in abdominal imaging). The two physicians were blinded to the surgical and pathological data of each patient, and the final conclusions were achieved through consensus decisions. The evaluated CT features consisted of qualitative and quantitative variables. Qualitative variables included location (cardia, fundus, body, or antrum), grow pattern (endophytic, exophytic, or mixed), contour (round, oval, or irregular), necrosis, calcification, surface ulceration, lymph node (LN), hemorrhage, peritumoral exudation, necrosis under the tumor wall, and intratumoral vessel. Quantitative variables included the CT attenuation value on unenhanced phase (CTU), arterial phase (CTA), and portal venous phase (CTV) imaging, the degree of enhancement on arterial phase (DEAP; DEAP = CTA – CTU) and portal venous phase (DEPP; DEPP = CTV − CTU) imaging, long diameter (LD), short diameter (SD), and the ratio of long diameter to short diameter (LD/SD). Lymph node was defined as positive when the short diameter of the lymph node was larger than 10 mm.

### Image preprocessing

Before image segmentation and feature extraction, it is necessary to preprocess images to reduce the data heterogeneity collected by different CT devices. First, the voxel size should be resampled to 1 × 1 × 1 mm^3^, and the anisotropic images should be homogenized to minimize the dependence of radiomics features on the image voxel size. The voxel intensity values were discretized using a fixed bin width of 25 HU with the aim of reducing image noise and standardizing the intensity to achieve a stable intensity resolution in all images. The images were normalized and the signal intensity was normalized to 1 to 500 HU to reduce the difference in signal intensity of images acquired by different machines. Z score was used to standardize the image gray value to reduce the influence of the inconsistency of image parameters on the variation of radiomics features.

### Image segmentation and radiomics feature extraction

**Fig. 2:**
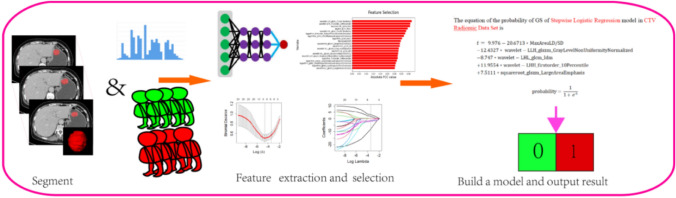
Flow diagram of this study. CT images were segmented on portal venous phase images. After preprocessing and feature selection, radiomics features, or clinical characteristics and CT features, were used to construct stepwise logistic regression and least absolute shrinkage and selection operator (LASSO) logistic regression models for differentiation of GSs from GISTs

Figure 2 depicts the workflow of this study. Open-source ITK-SNAP software (version 2.2.0; http://www.itk-snap.org) was used for image segmentation. Portal venous phase images were used for image segmentation in this study since the portal venous phase had a better performance than unenhanced and arterial phases in identifying the lesion from the surrounding normal tissue. Region of interests (ROIs) were drawn manually along the margin of tumors in a slice-by-slice manner by two radiologists (with 2 and 4 years of experience in abdominal imaging). The radiomics characteristics were then extracted using Pyradiomics (version 3.0.0; http://www.radiomics.io/pyradiomics.html).

A total of 1223 radiomics features were extracted from the tumor region of interests (ROIs) drawn on the portal venous phase imaging: (1) 19 first-order features; (2) 26 size- and shape-related features; (3) 24 Gy level co-occurrence matrix (GLCM) features; (4) 14 Gy level dependence matrix (GLDM) features; (5) 16 Gy level run length matrix (GLRLM) features; (6) 16 Gy level size zone matrix (GLSZM) features; (7) 5 neighboring gray tone difference matrix (NGTDM) features; and (8) 1103 features after transformations including square, square root, exponential, logarithm, wavelet, and Laplacian of Gaussian.

To assess inter- and intra-observer reproducibility, 40 ROIs of 2 tumors delineated by 2 radiologists were chosen at random. Inter- and intra-class correlation coefficients (ICCs) were used to evaluate the stability and reproducibility in radiomics feature extraction. The intra-observer ICCs were calculated based on the twice feature extractions by one radiologist. The inter-observer ICCs were evaluated based on the first extracted features by one radiologist and those by another radiologist. The feature with ICC > 0.75 was deemed to have a good repeatability or reproducibility. After inter- and intra-observer analyses, a total of 968 features with ICC > 0.75 were applied for subsequent feature selection.

### Feature selection and model building

First, feature dimensionality reduction was performed before model building. The Pearson correlation coefficient (PCC) between two features was calculated for each pair of features in the non-radiomics and radiomics datasets. These two features were considered to be highly correlated if the PCC larger than 0.8, and one of them was selected for deletion. Then we calculated the PCCs between features and response variables (GSs or GISTs) and selected the 20 features with the largest PCCs.

These 20 features selected from the clinical and radiomics features were used to build 2 kinds of models: a stepwise logistic regression model and a LASSO-logistic regression model. The two models were constructed using data from the training dataset and were tested using the test dataset.

#### Stepwise logistic regression

Logistic regression models were plotted using the R package “glm”. Initially, all 20 variables were input into the model and the variables with the largest *p* value were eliminated in each cycle, with the variables with *p* values less than 0.1 being continually entered into the univariate analysis until the variables no longer changed.

#### LASSO-logistic regression

The LASSO algorithm was applied with the R package “glmnet”. The most useful predictive features were selected using the LASSO method. Briefly, the optimized hyper-parameter λ was determined using tenfold cross-validation via the minimum criteria. Then the features with non-zero coefficients were selected based on the determined optimal λ.

##### Statistical analysis

All statistical analyses were performed using R software (version 3.6.3; http://www.Rproject.org). Continuous variables are presented as mean ± standard deviation. Student’s *t* test or the Mann–Whitney *U* test was used to compare continuous variables. Categorical variables are expressed as frequency (percentage) and were analyzed using the Chi-square or Fisher’s exact test. Sensitivity, specificity, and accuracy were calculated using the ‘caret’ package in R. ROC curve analysis was performed with the ‘pROC’ package to evaluate the diagnostic efficacy of the models. Delong’s test was used to compare the AUC values between the different methods. A two-sided *p* value of < 0.05 was considered statistically significant.

## Results

### Clinical baseline characteristics and CT findings

Two hundred forty-three patients with tumors (forty-three GSs and two hundred GISTs) from Center 1 and fifty-nine patients with tumors (six GSs and fifty-threeGISTs) from Center 2 were included in our series. The clinical baseline characteristics and CT findings of the 302 patients in the training (*n = *211) and validation (*n = *91) cohorts are listed in Table 1. For both training and validation sets, the number of underlying diseases, lesion location, necrosis, and necrosis under the tumor wall were significant variables in the univariate analysis between the two tumor types, while growth pattern was significantly different in the training cohort. Although patients with GIST were older than those with GS both in training and validation cohorts, the differences were not statistically significant. There were no significant differences in any of the clinical and CT parameters between the two datasets (all* p* > 0.05), which indicates that the patient allocation to the two datasets was well balanced. The detailed results are summarized in Table 1.

**Table 1:** Clinical baseline characteristics and CT findings in training and validation cohorts

	Training cohort(*n = *211)			Validation cohort (*n*91)			
	GS(*n = *34)	GIST(*n = *177)	*P1* value^a^	GS(*n = *15)	GIST(*n = *76)	*P2* value^b^	*P3* value^c^
Clinical characteristics							
Gender (male/female)	(11/23)	(91/86)	**0.042**	(5/10)	(40/36)	0.259	0.900
Age (Mean ± SD)	56.38 ± 10.05	59.70 ± 11.89	0.129	56.80 ± 11.32	59.22 ± 11.12	0.444	0.813
Symptom	(17/59)	(17/118)	0.079	(6/9)	(53/23)	**0.039**	0.968
Tumor makers	(8/46)	(26/131)	0.833	(4/11)	(20/56)	1.000	1.000
Number of underling disease s(0/1/2/ ≥ 3)	(28/4/2/0)	(106/46/18/7)	**0.002**	(12/3/0/0)	(43/23/8/2)	**0.004**	0.546
CT features							
Location (cardia/fundus/body/antrum)	(0/2/20/12)	(6/57/97/17)	**0.000**	(0/0/11/4)	(3/25/42/6)	**0.009**	0.911
Growth pattern (endophytic/ exophytic/mixed)	(4/18/12)	(66/67/44)	**0.015**	(3/7/5)	(30/33/13)	0.209	0.466
Contour (round/oval/irregular)	(9/16/16)	(51/46/80)	0.064	(7/6/2)	(30/16/30)	0.092	0.307
Necrosis (yes/no)	(3/31)	(95/82)	**0.000**	(1/14)	(33/43)	**0.000**	0.802
Calcification (yes/no)	(0/34)	(28/149)	**0.027**	(0/15)	(13/63)	0.185	0.855
Surface ulceration (yes/no)	(3/31)	(37/140)	0.149	(2/13)	(16/60)	0.740	0.874
LN (yes/no)	(6/28)	(15/162)	0.186	(2/13)	(6/70)	0.614	0.834
Hemorrhage (yes/no)	(0/34)	(2/175)	1.000	(0/15)	(1/75)	1.000	1.000
Peritumoral exudation (yes/no)	(0/34)	(5/172)	1.000	(0/15)	(1/75)	1.000	0.782
Necrosis under the tumor wall (yes/no)	(2/32)	(69/108)	**0.000**	(2/13)	(29/47)	**0.017**	1.000
Intratumoral vessel (yes/no)	(5/29)	(35/142)	0.635	(2/32)	(16/60)	0.740	0.874
CTU(HU)	34.80 ± 5.00	34.40 ± 7.10	0.755	32.33 ± 4.34	35.43 ± 8.70	0.183	0.619
CTA(HU)	56.47 ± 12.85	58.51 ± 17.31	0.514	55.71 ± 13.94	57.37 ± 13.10	0.672	0.270
CTV(HU)	71.20 ± 15.63	71.90 ± 18.10	0.826	68.45 ± 17.85	75.04 ± 13.31	0.179	0.307
DEAP(HU)	21.67 ± 10.80	24.11 ± 16.61	0.278	20.28 ± 12.48	25.05 ± 12.05	0.178	0.158
DEPP(HU)	36.38 ± 14.39	37.51 ± 18.41	0.735	33.03 ± 17.57	42.71 ± 11.37	0.054	0.221
LD (mm)	28.79 ± 13.74	44.83 ± 34.94	**0.000**	24.40 ± 10.83	50.87 ± 40.60	**0.000**	0.346
SD (mm)	28.50 ± 12.23	35.26 ± 24.42	**0.000**	25.67 ± 13.32	37.80 ± 28.41	**0.015**	0.594
LD/SD	1.02 ± 0.21	1.26 ± 0.25	**0.000**	1.00 ± 0.18	1.28 ± 0.24	**0.000**	0.813

*GIST* gastrointestinal stromal tumor; *GS* gastric schwannoma; *CTU/CTA/CTV* the CT attenuation value of unenhancement phase/arterial phase/portal venous phase; *DEAP* the CT attenuation value of arterial phase-unenhanced phase; *DEPP* CT attenuation value of portal venous phase-unenhanced phase; *LD* long diameter; *SD* short diameter

*P1* value^a^: comparison of GSs and GISTs in training cohort

*P2* value^b^: comparison of GSs and GISTs in validation cohort

*P3* value^c^: comparison of patients in training cohort and patients in validation cohort

*P* value written in bold indicates a significant difference

### Feature selection and model building

**Fig. 3:**
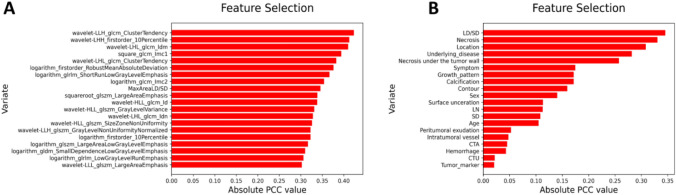
Feature selection by calculation of the Pearson correlation coefficients (PCCs) before model building. The 20 features with the largest PCC were selected as input indexes to build models using features from non-radiomics dataset (**A**) and radiomics dataset (**B**)

The clinical features, CT features, and radiomics features were reduced in dimension by eliminating those features with PCC values of more than 0.8 with another feature. The PCC values (between features and GSs) in the 235 remaining radiomics features and 24 clinical characteristics and CT features were then calculated, and the 20 features with the largest PCC values were selected. The 20 features selected in the 2 datasets are shown in Fig. 3. Among the selected radiomics features, wavelet-LLH_glcm_ClusterTendency had the largest PCC, and wavelet-LLL_glszm_LargeAreaEmphasis the smallest PCC. Among the clinical features, LD/SD showed the largest PCC and tumor marker the lowest PCC.

#### Stepwise logistic regression model

Table 2 presents the five radiomics features (and their coefficients) with *p* < 0.05 selected from the training dataset according to the stepwise logistic regression model. These features included one from size- and shape-related features, two from GLSZM, and one from GLCM. The wavelet-LHH_firstorder_10Percentile with the largest β (SE) value (11.9554) increased the likelihood of GSs diagnosis rather than GISTs. However, MaxAreaLD/SD with a β (SE) value of -20.6713 provided the greatest contribution to the prediction of GIST.

**Table 2:** The selected features and relevant coefficients of the stepwise logistic regression model in radiomics dataset

Variate	β (SE)	β(SE)[0.025	0.975]	*P* value
Constant	9.976	3.728	16.224	**0.002**
MaxAreaLD/SD	− 20.6713	− 28.626	− 12.717	**0.000**
Wavelet-LLH_glszm_ GrayLevelNonUniformityNormalized	− 12.4327	− 19.886	− 4.979	**0.001**
wavelet-LHL_glcm_Idm	− 8.747	− 14.74	− 2.754	**0.004**
wavelet-LHH_firstorder_10Percentile	11.9554	6.996	16.915	**0.000**
squareroot_glszm_LargeAreaEmphasis	7.5111	3.151	11.871	**0.001**

*P* value written in bold indicates a significant difference

Six clinical and CT features (sex, LD/SD, location, growth pattern, necrosis, and underlying disease) were selected by the stepwise logistic regression model, as shown in Table 3. A location in the body and antrum had the highest association with a diagnosis of GS, while a large LD/SD value was most associated with a finding of GIST.

**Table 3:** The selected features and relevant coefficients of the Stepwise Logistic Regression model in non-radiomic dataset

Variate	β (SE)	β(SE)[0.025	0.975]	*P* value
Constant	− 1.5608	− 4.398	1.276	0.281
Sex	− 1.1934	− 2.343	− 0.044	**0.042**
LD/SD	− 8.3933	− 14.564	− 2.223	**0.008**
Location	5.1244	1.993	8.256	**0.001**
Growth pattern	2.1954	0.704	3.687	**0.004**
Necrosis	− 2.7189	− 4.11	− 1.328	**0.000**
Underlying disease	− 5.1407	− 8.399	− 1.883	**0.002**

*LD:* long diameter; *SD:* short diameter

*P* value written in bold indicates a significant difference

The equation for calculating the probability of GS in the stepwise logistic regression model for radiomics features was:

where  

$$\begin{gathered} {\text{probability = }}\frac{1}{{1 + e^{{ - t}} }} \hfill \\ t = 9.976 - 20.6713*{\text{MaxAreaLD}}/{\rm{SD}} \hfill \\ \quad - 12.4327{{*}}~{\text{wavelet}} - {\text{LLH}}\_{\rm{glszm}}\_{\text{Gray}} {\rm{Level}} {\text{NonUniformity}} {\rm{Normalized}} \hfill \\ \quad - 8.747*{\text{wavelet}} - {\rm{LHL}}\_{\text{glcm}}\_{\rm{Idm}} \hfill \\ \quad + 11.9554{\text{*wavelet}} - {\rm{LHH}}\_{\text{firstorder}}\_10{\rm{Percentile}} \hfill \\ \quad + 7.5111*{\text{squareroot}}\_{\rm{glszm}}\_{\text{LargeAreaEmphasis}} \hfill \\ \end{gathered}$$

For the non-radiomics dataset, the equation for calculating the probability of GS was

$${\text{probability = }}\frac{1}{{1 + {\text{e}}^{{\rm{ - t}}} }}$$

Where

$$\begin{gathered} {\text{t}} = - 1.5608 - 1.1934*{\text{Sex}} - 8.3933*\frac{{{\text{LD}}}}{{{\rm{SD}}}} + 5.1244*{\text{Location}} \hfill \\ \quad \quad + 2.1954*{\rm{Growthpattern}} - 2.7189*{\text{Necrosis}} - 5.1407*{\rm{Underlyingdisease}} \hfill \\ \end{gathered}$$

#### LASSO-logistic regression model

**Fig. 4:**
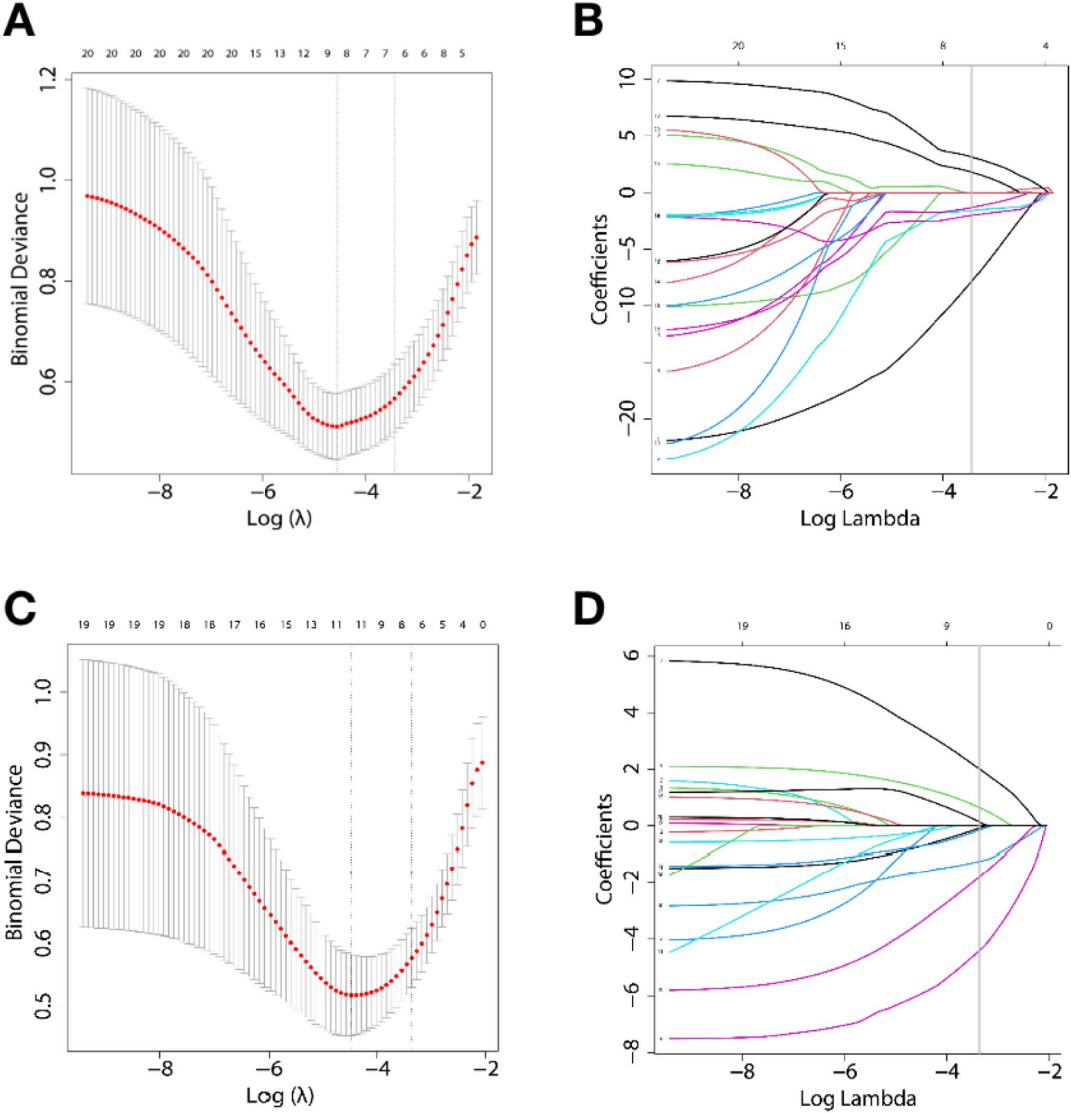
Feature selection via LASSO-logistic regression. Radiomics feature selection (**A**, **B**). Selection of the tuning parameter (λ) using tenfold cross-validation and a minimum criterion. A λ value of 0.011 with log (λ) =  − 4.50 was selected. A coefficient profile is plotted against the log (λ) sequence using tenfold cross-validation. A vertical line is drawn at the value selected, which resulted in ten non-zero coefficients. Clinical and CT features selection (**C**, **D**). The optimal λ value of 0.037 with log (λ) =  − 3.31 was retained using tenfold cross-validation. Eight non-zero coefficients were selected finally

The results of the LASSO**-**logistic regression using features from non-radiomics dataset or radiomics dataset are summarized in Tables 4 and 5 and Fig. 4. The LASSO method selected nine radiomics features and eight clinical and CT features for input into the regression algorithm. High MaxAreaLD/SD and LD/SD yielded the largest coefficients supporting the identification of GISTs in the two datasets. Supplemental Table S1 presents the five selected radiomics features with *p* < 0.05 and compares them between GSs and GISTs in the training dataset.

**Table 4:** The selected features and relevant coefficients of LASSO-logistic regression model in radiomics dataset

Variate	β (SE)	β(SE)[0.025	0.975]	*P* value
Constant	8.5564	− 0.725	17.838	0.071
MaxAreaLD/SD	− 17.0994	− 24.064	− 10.135	**0.000**
wavelet-LHL_glcm_ClusterTendency	− 0.8538	− 6.172	4.465	0.753
wavelet-LHL_glcm_Idm	− 3.8902	− 12.601	4.82	0.381
wavelet-LHH_firstorder_10Percentile	5.6835	2.267	9.1	**0.001**
square_glcm_Imc1	− 3.2946	− 10.537	3.948	0.373
squareroot_glszm_LargeAreaEmphasis	3.886	0.014	7.758	**0.049**
logarithm_firstorder_RobustMeanAbsoluteDeviation	− 1.238	− 7.659	5.183	0.706
logarithm_glrlm_ShortRunLowGrayLevel Emphasis	− 2.8064	− 9.113	3.5	0.383
logarithm_glszm_LargeAreaLowGrayLevelEmphasis	1.4232	− 3.513	6.359	0.572

*P* value written in bold indicates a significant difference

**Table 5:** The selected features and relevant coefficients of LASSO-logistic regression in non-radiomics dataset

Variate	β (SE)	β(SE)[0.025	0.975]	*P* value
Constant	− 1.6349	− 4.569	1.299	0.275
Sex	− 1.3814	− 2.613	− 0.149	**0.028**
LD/SD	− 8.0618	− 14.016	− 2.108	**0.008**
Location	5.2375	2.028	8.447	**0.001**
Growth pattern	2.0087	0.489	3.529	**0.010**
Necrosis	− 1.9847	− 3.893	− 0.077	**0.041**
LN	1.9776	− 0.094	4.049	0.061
Necrosis under the tumor wall	− 1.5074	− 3.805	0.79	0.199
Underlying disease	− 5.2904	− 8.755	− 1.826	**0.003**

*LD/SD:* the ratio of long diameter to short diameter; *LN:* lymph node

*P* value written in bold indicates a significant difference

The probability of GS according to the radiomics datasets was equal to

$$\text{probability = }\frac{1}{1+{e}^{{-t}}}$$

where

$$\begin{gathered} t = 8.5564 - 17.0994 *{\text{ MaxAreaLD/SD}} \hfill \\ \quad -0.8538 * {\text{wavelet-LHL}}\_{\rm{glcm}}\_{\text{ClusterTendency}} \hfill \\ \quad - 3.8902*{\text{wavelet-LHL}}\_{\text{glcm}}\_{\rm{Idm}} \hfill \\ \quad +5.6835* {\text{wavelet-LHH}}\_{\rm{firstorder}}\_{\text{10Percentile}} \hfill \\ \quad -3.2946* {\text{square}}\_{\rm{glcm}}\_{\text{Imc1}} \hfill \\ \quad + 3.886*{\rm{squareroot\_glszm\_LargeAreaEmphasis}} \hfill \\ \quad -1.238* {\text{logarithm}}\_{\rm{firstorder}}\_{\text{RobustMeanAbsoluteDeviation}} \hfill \\ \quad - {\rm{2.8064*logarithm}}\_{\text{glrlm}}\_{\rm{ShortRunLowGrayLevelEmphasis}} \hfill \\ \quad + 1.4232*{\text{logarithm}}\_{\rm{glszm}}\_{\text{LargeAreaLowGrayLevelEmphasis}} \hfill \\ \end{gathered}$$

For the non-radiomics dataset, the equation for the probability of GS was

$${\text{probability = }}\frac{1}{{1 + e^{{\rm{ - t}}} }}$$

$$\begin{gathered} {\text{t = }} - \,\,1.6349 - 1.{\text{3814 * Sex}} - 8{\text{.0618 * LD/SD + 5}}{\text{.2375 * Location}} \hfill \\ \quad +\, 2.0087 * {\text{Growth pattern }} - 1{\text{.9847 * Necrosis + 1}}{\text{.9776 * LN }} \hfill \\ \quad - 1.{\text{5074 * Necrosis under the tumor wall - 5}}{\text{.2904 * Underlying disease}} \hfill \\ \end{gathered}$$

### Diagnostic performance analysis

**Fig. 5:**
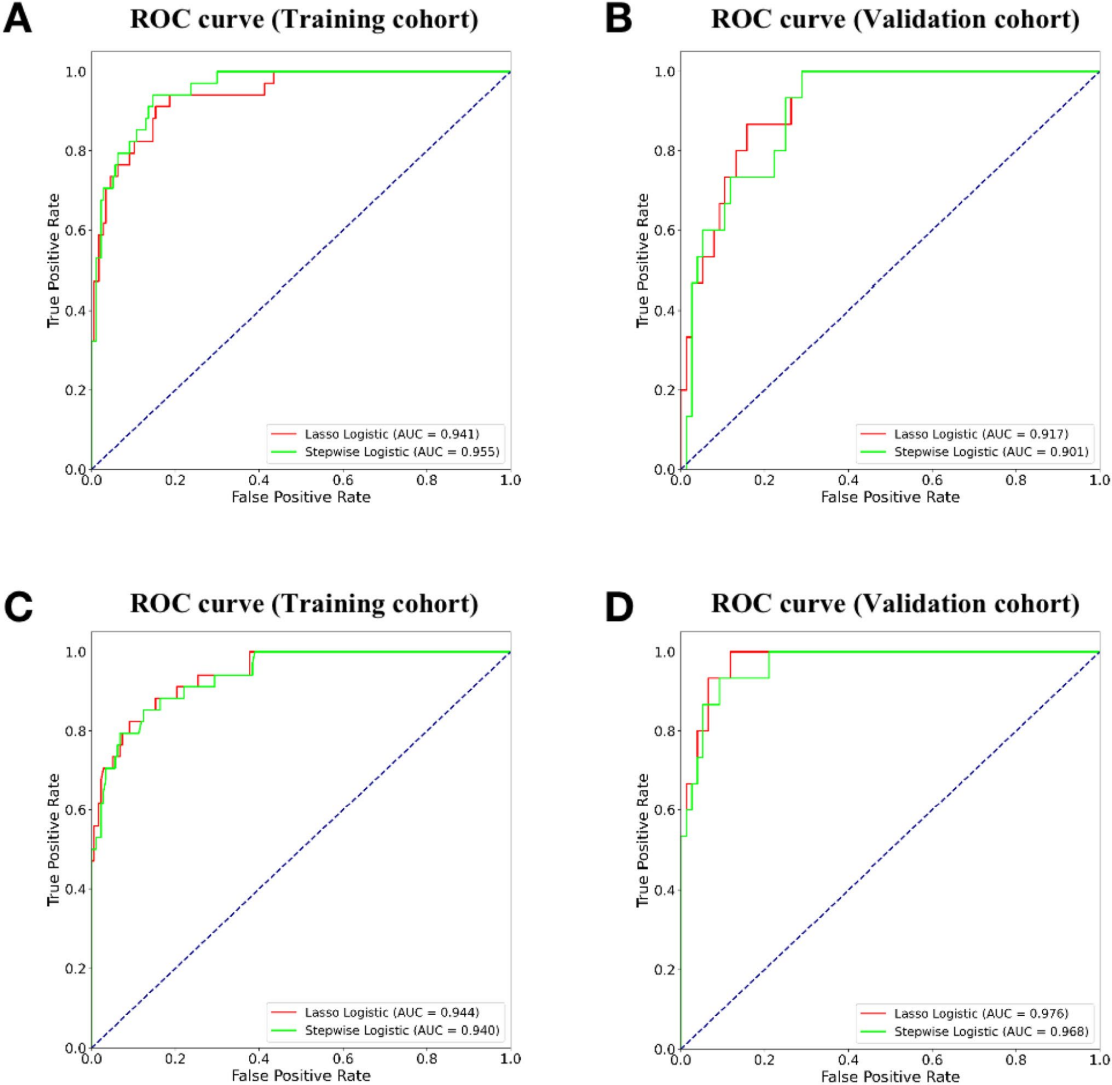
Receiver operating characteristic (ROC) curves of two models for differentiating GS and GIST. All the AUCs of the two models using features from radiomics dataset (**A**, **B**) and non-radiomics dataset (**C**, **D**) were above 0.900

The diagnostic efficacy of the two radiomics feature models in the training and validation sets is summarized in Table 6. The stepwise logistic regression model applied to the training dataset yielded sensitivity, specificity, accuracy, and AUC of 94.1%, 85.3%, 86.7%, and 0.955, respectively, whereas the LASSO-logistic regression model yielded sensitivity, specificity, accuracy, and AUC of 91.2%, 84.7%, 85.8%, and 0.941. Supplemental Table S2 shows the diagnostic performance results for the features from non-radiomics dataset. The ROC curves of the two models applied to the training and validation datasets are plotted in Fig. 5. We found that the AUC values of the two kinds of models were larger than 0.900 for both datasets.

**Table 6:** The diagnostic performance analysis in radiomics dataset

Cohort	Model	Sensitivity (%)	Specificity (%)	Accuracy (%)	AUC
Training(*n = *211)	Stepwise logistic regression	94.1	85.3	86.7	0.955
	LASSO-logistic regression	91.2	84.7	85.8	0.941
Validation(*n = *91)	Stepwise logistic regression	93.0	71.1	75.8	0.901
	LASSO-logistic regression	95.6	71.1	72.8	0.917

*AUC:* area under the curve

### Delong’s test analysis

Delong’s test was independently implemented in the training and validation datasets. We compared the diagnostic efficiency differences between the two data sets within the same model (stepwise logistic regression or LASSO-logistic regression). Table 7 presents the Delong’s test analysis results. There were no significant differences in predictive performance between features from non-radiomics and radiomics datasets, regardless of which model was used (all *p* > 0.05).

**Table 7:** The Delong’s test analysis of the two models in different dataset

Cohort	Model	Dataset	AUC	*P* value
Training (*n = *211)	Stepwise logistic regression	Radiomics dataset	0.955	0.493
		Combination dataset	0.940	
	LASSO-logistic regression	Radiomics dataset	0.941	0.902
		Combination dataset	0.944	
Validation (*n = *91)	Stepwise logistic regression	Radiomics dataset	0.901	0.089
		Combination dataset	0.968	
	LASSO-logistic regression	Radiomics dataset	0.917	0.079
		Combination dataset	0.976	

*AUC:* area under the curve

Supplemental Tables S3 and S4 separately summarize the Delong’s test results between the stepwise logistic regression model and LASSO-logistic regression model in the two datasets.

## Discussion

While a number of studies have investigated the differentiation of GSs from gastric stromal tumors using CT images, we believe this study is the first attempt to apply radiomics to this problem. In our study, 211 patients with either GISTs or GSs from Center 1 were assigned to training cohort and 91 patients from Center 2 to validation cohort. Univariate analysis showed that the clinical and CT parameters were well balanced between the two cohorts.

In our study, 1223 extracted radiomics features and 24 clinical and CT characteristics were used to build stepwise logistic regression and LASSO-logistic regression models. A total of eight models using two kinds of algorithms were developed, and the AUC values attained were above 0.9 for both datasets with both radiomics features and clinical and CT features. Although a Delong’s test revealed no significant differences in predictive performance between the non-radiomics dataset and radiomics dataset, we still suggest that the radiomics-based model shows promise, with comparable predictive performance to senior physicians in the differentiation of GS from GIST.

Five radiomics features were selected by the stepwise logistic regression model as useful parameters to differentiate GS from GIST, and three of these features were picked in the LASSO-logistic regression.

MaxAreaLD/SD is a shape-based feature, equal to the ratio of the major axis length to the least axis length, which is similar to the LD/SD feature manually extracted from the CT imaging. In this study, the MaxAreaLD/SD and LD/SD values were higher for GISTs than for GSs. High MaxAreaLD/SD from the radiomics features and LD/SD clinical values were of greater benefit to identification of GISTs rather than GSs, which reveals that GISTs, as a potentially malignant entity, tended to grow at different rates in all directions and had a more irregular shape than GSs.

The features wavelet-LLH_glszm_GrayLevelNonUniformityNormalized, wavelet-LHL_glcm_Idm, and wavelet-LHH_firstorder_10Percentile are wavelet texture features. Wavelet features are high-order features that not only reflect the distribution characteristics of space and frequency, but also reflect the heterogeneity of the tumor’s microenvironment, and they are known to improve the diagnostic performance of radiomics models (Zhou et al. 2020). GrayLevelNonUniformityNormalized and glszm_LargeAreaEmphasis are both GLSZM characteristics. GLSZM features are used to calculate the number of connections of voxels with the same gray value in the image. Wavelet_HHL_glszm_GrayLevelNonUniformity refers to the non-uniformity of the gray levels; the lower the value is, the more uniform the gray level and the lower the heterogeneity of the image. GSs showed lower wavelet_HHL_glszm_GrayLevelNonUniformity values than GISTs, which indicated that GSs had more homogeneous parenchyma. Firstorder_10Percentile describes the distribution of voxel intensities in low-density regions. The lower values found in GISTs may be due to their susceptibility to necrosis and cysts. GLCM features can reflect the distribution of the gray levels of two pixels in a specific direction and distance, and are most widely used to evaluate tumor heterogeneity. The glcm_Idm feature reflects local changes in the image texture. If different areas of the image texture are more uniform, the inverse difference will be larger, whereas otherwise it will be smaller. Large glcm_Idm values in GSs indicate a more uniform nature.

Among the clinical and CT features, sex, LD/SD, location, growth pattern, necrosis, and number of underlying diseases were found to be significant features for the differentiation of GSs from GISTs. GS was more predominantly found in female patients than GIST, which is consistent with the previous studies of Ji et al. (2015), but different to the finding of Xu et al. (2022). We found that GSs tended to grow in the gastric body and antrum, whereas gastric GISTs were often seen in the body and the fundus, which is similar to the findings in Xu et al. (2022) and our previous reports (Wang et al. 2021). An exophytic or mixed growth pattern found in GS in several previous studies (Choi et al. 2012; He et al. 2017; Xu et al. 2022) is also in agreement with our findings. Our study also revealed necrosis to be a significant CT feature suggesting GIST rather than GS, which is in line with the study of He et al. (He et al. 2017). The lack of necrosis in schwannomas may be a result of the slower growth rate of GS compared with GIST (Choi et al. 2014). To our surprise, patients with GIST tended to have more underlying diseases than those with GS, which has not been reported before. The underlying diseases included hypertension, diabetes, cardiovascular disease, cerebrovascular disease, chronic obstructive pulmonary disease, and chronic kidney diseases. The reason for this may be related to the fact that our patients with GIST were older than those with GS, and the possibility of suffering from underlying diseases may increase accordingly. We also speculate that patients with underlying diseases may be more likely to be affected by malignant tumors than benign ones, although this hypothesis still requires more cases and studies to confirm it.

This study has several limitations. First, the sample size is relatively small, especially for GSs. Our series was collected from two hospitals and was acquired using two different CT scanners, which may have resulted in data heterogeneity. Second, the radiomics features were only extracted from portal venous phase images. Radiomics information from unenhanced and arterial phase images was not applied to optimize the performance of the models. Hence, we intend to build radiomics models using features from all three phases in future work. Finally, the models built from CT features assessed by two associate chief radiologists showed good performance, but we did not make a comparison with predictive performance assessed by junior radiologists. It is possible that poorer performance may be shown assessed by junior radiologists, highlighting the excellence of the radiomics methods.

## Conclusion

This preliminary study built radiomics-based models to explore associations between radiomics features and GSs and GISTs. Our study showed that a radiomics model applied to CE-CT images had comparable predictive performance to senior radiologists in the differentiation of GSs from GISTs.

### Supplementary Information

Below is the link to the electronic supplementary material.

Supplementary file1 (DOCX 16 KB)

## Data Availability

The datasets used and/or analyzed during the current study are available from the corresponding author on reasonable request.

## Abbreviations

AUC: Area under the curve
CE-CT: Contrast-enhanced computed tomography
CTU/CTA/CTV: The CT attenuation value in unenhanced phase, arterial phase, and portal venous phase
DEAP/DEPP: Degree of enhancement on arterial phase and portal venous phase
GIST: Gastrointestinal stromal tumor
GLCM: Gray level co-occurrence matrix
GLDM: Gray level dependence matrix
GLRLM: Gray level run length matrix
GLSZM: Gray level size zone matrix
GS: Gastric schwannoma
ICC: Inter-and intra-class correlation coefficient
LASSO: Least absolute shrinkage and selection operator
LD: Long diameter
LD/SD: Ratio of long diameter to short diameter
LN: Lymph node
NGTDM: Neighboring gray tone difference matrices matrix
ROI: Region of interest
PCC: Pearson correlation coefficient
ROC: Receiver operating characteristics
SD: Short diameter
