# Supplementary material for: Radiomics analysis of contrast-enhanced computerized tomography for differentiation of gastric schwannomas from gastric gastrointestinal stromal tumors
Source: J Cancer Res Clin Oncol. 2024 Feb 9;150(2):87. doi: 10.1007/s00432-023-05545-w (PMC10858083; doi:10.1007/s00432-023-05545-w)
Supplement: Supplementary file 1 — Supplementary file1 (DOCX 16 KB) [file 432_2023_5545_MOESM1_ESM.docx]

**Supplementary files**

**Table S1** Five selected radiomics features of GS and GIST in training cohort

| **Variate** | **GS (n﹦34)** | **GIST (n﹦177)** | ***P* value** |
| --- | --- | --- | --- |
| MaxAreaLD/SD | 1.02±0.21 | 1.26±0.25 | **0.000** |
| Wavelet-LLH_glszm_  GrayLevelNonUniformityNormalized | 0.06±0.03 | 0.08±0.03 | **0.000** |
| wavelet-LHL_glcm_Idm | 4.72±0.77 | 5.36±0.46 | **0.000** |
| wavelet-LHH_firstorder_10Percentile | 0.94±0.59 | 0.43±0.38 | **0.000** |
| squareroot_glszm_LargeAreaEmphasis | 1.02±1.06 | 2.23±1.93 | **0.001** |

*P* value written in bold indicates a significant difference

**Table S2** The diagnostic performance analysis in non-radiomic dataset

| **Cohort** | **Model** | **Sensitivity** | **Specificity** | **Accuracy** | **AUC** |
| --- | --- | --- | --- | --- | --- |
| Training(n=211) | Stepwise Logistic Regression | 85.3% | 87.6% | 87.2% | 0.940 |
|  | Lasso-Logistic Regression | 82.4% | 91.0% | 89.6% | 0.944 |
| Validation((n=91) | Stepwise Logistic Regression | 93.3% | 90.8% | 91.2% | 0.968 |
|  | Lasso-Logistic Regression | 100.0% | 88.2% | 90.1% | 0.976 |

*AUC* area under the curve

**Table S3 The Delong’s test result of the two models in radiomic dataset**

| **Cohort** | **Model** | **AUC** | ***P* value** |
| --- | --- | --- | --- |
| Training(n=211) | Stepwise Logistic Regression | 0.955 | 0.138 |
|  | Lasso-Logistic Regression | 0.941 |  |
| Validation(n=91) | Stepwise Logistic Regression | 0.901 | 0.436 |
|  | Lasso-Logistic Regression | 0.917 |  |

*AUC* area under the curve

**Table S4 The Delong’s test result of the two models in non-radiomic dataset**

| **Cohort** | **Model** | **AUC** | ***P* value** |
| --- | --- | --- | --- |
| Training(n=211) | Stepwise Logistic Regression | 0.940 | 0.436 |
|  | Lasso-Logistic Regression | 0.944 |  |
| Validation(n=91) | Stepwise Logistic Regression | 0.968 | 0.416 |
|  | Lasso-Logistic Regression | 0.917 |  |

*AUC* area under the curve
